# 984. Detection of Mycobacterial Species from Specimens Submitted for Metagenomic Analysis

**DOI:** 10.1093/ofid/ofad500.039

**Published:** 2023-11-27

**Authors:** Joseph F John, Michael G Schmidt, Courtney L Jarvis, Niccole Diaz, Rick Martin

**Affiliations:** Low Country Infectious Diseases, PA, Charleston, South Carolina; Medical University of SC, Charleston, South Carolina; MicroGenDX, Lubbock, Texas; MicroGenDx, Lubbock, Texas; MicroGenDX, Lubbock, Texas

## Abstract

**Background:**

A national U.S. epidemic of non-tuberculous mycobacterial (NTM) infections is occurring at rates unseen over earlier decades, resulting in substantial numbers of pathogenic sequelae. Culture of mycobacteria is laborious, often requiring weeks for cultivation and speciation. Next generation sequencing (NGS) to detect NTM from clinical specimens has advantages over culture methods. From January 2018 to June 2022 we queried a large national NGS database to detect NTM rates and species.

**Methods:**

DNA was isolated from 431,716 patient specimens collected from a variety of body sites: sputum, urine, sinus, wound or an unspecified swabs. Employing an Illumina’s 16S and ITS targeted-gene, deep sequencing approach, bacterial and/or fungal species resident in the specimen were determined. The identity and relative abundance of mycobacterial DNA recovered for each specimen were reported by species as the primary outcomes.

**Results:**

Of the 431,716 specimens analyzed in this 4.5 year retrospective study, 0.001% (468) contained mycobacterial DNA. Forty different species of mycobacteria were detected with the most common frequency including: *M abscessus*, 40%; *M mucogenicum*, 13%; *M. tuberculosis*, 8.5% and *M immunogenum* 4.6%. *M. avian and M. intracellulare* were each detected in only 4% of the positive NTM specimens. 15% were other NTM species and 15% were not speciated NTM. Between 2018-2022 of the 468 NTM, the annual number of *M*. *abscessus* was 38, 53, 41 and 41, respectively. The concentration or relative abundance of mycobacterial DNA within each specimen varied from 4 to 100% of the total bacterial DNA recovered.

**Conclusion:**

Over 4.5 years , NGS performed at a national molecular diagnostic laboratory detected 40 NTM species with a predominant fraction as *M. abscessus (40%), supporting the clinical concern for an* epidemic increase in *M. abscessus* infections during the last half decade. These data support the view that NGS metagenomics can uncover NTM epidemiological trends and implicate NTM infections.

**Disclosures:**

**Joseph F. John, Jr., MD, FACP, FIDSA**, MicroGenDx: Advisor/Consultant **Michael G. Schmidt, PhD**, MicroGenDx: Advisor/Consultant **Courtney L. Jarvis, PhD**, MicroGenDX: Employee **Niccole Diaz, BS**, MicroGenDX: Advisor/Consultant **Rick Martin, BS**, MicroGenDX: Ownership Interest

